# The Activity of Surface Electromyographic Signal of Selected Muscles during Classic Rehabilitation Exercise

**DOI:** 10.1155/2016/4796875

**Published:** 2016-04-19

**Authors:** Jinzhuang Xiao, Jinli Sun, Junmin Gao, Hongrui Wang, Xincai Yang

**Affiliations:** ^1^College of Electronic and Information Engineering, Hebei University, Baoding, Hebei 071000, China; ^2^Healthy Rehabilitation Section, Affiliated Hospital of Hebei University, Baoding, Hebei 071000, China

## Abstract

*Objectives*. Prone bridge, unilateral bridge, supine bridge, and bird-dog are classic rehabilitation exercises, which have been advocated as effective ways to improve core stability among healthy individuals and patients with low back pain. The aim of this study was to investigate the activity of seven selected muscles during rehabilitation exercises through the signal of surface electromyographic.* Approaches*. We measured the surface electromyographic signals of four lower limb muscles, two abdominal muscles, and one back muscle during rehabilitation exercises of 30 healthy students and then analyzed its activity level using the median frequency method.* Results*. Different levels of muscle activity during the four rehabilitation exercises were observed. The prone bridge and unilateral bridge caused the greatest muscle fatigue; however, the supine bridge generated the lowest muscle activity. There was no significant difference (*P* > 0.05) between left and right body side muscles in the median frequency slope during the four rehabilitation exercises of seven muscles.* Conclusions*. The prone bridge can affect the low back and lower limb muscles of most people. The unilateral bridge was found to stimulate muscles much more active than the supine bridge. The bird-dog does not cause much fatigue to muscles but can make most selected muscles active.

## 1. Introduction

Rehabilitation exercise has received more and more attention, including the general public, athletes, rehabilitation therapists, and the scientific researcher, when the people's demands for health are upgraded gradually. We know that core stability not only has significance for rehabilitation of patients with low back pain, but also can prevent strain of lumbar muscles and other chronic diseases in healthy people, which is concluded through communicating with rehabilitation therapists and looking up correlation literatures. Based on previous researches, there were many published results about the importance of the abdominal and back muscles [1–13]. Although Ekstrom et al. [13] found that bird-dog exercise can strengthen gluteus maximum muscle and side bridge can be used for strengthening the gluteus medius muscle during the 9 rehabilitation exercises and there were some investigations regarding the activity of lower limbs [14, 15] which were not about classic rehabilitation exercises, there are no more studies about calf muscles during rehabilitation exercises. In this research, we focused on the low back muscles and some selected lower limb muscles (thigh and calf muscles) during classic rehabilitation exercises.

According to the previous literatures [13, 16–18], the exercises of elbow-toe, back bridge, hand-knee, and so on in stable plane which have been analyzed using the sEMG signal. They had found that these rehabilitation exercises had some effect on our lumbar and back muscles to train core stability through letting healthy people do these exercises. Imai et al. [18] compare trunk stabilization (SE, front plank, back bridge, quadruped exercise, and side bridge) to conventional exercises (CE, sit-up-1, sit-up-2, back extension-1, and back extension-2) which were in stable plane, and they found that SE has apparent training effects on static and dynamic balance. However, rehabilitation exercises have been much more adequate than ever because they have made lots of improvements on the traditional rehabilitation exercises. Swiss ball [4–10, 19] has been applied in research to analyze the influence of performing the rehabilitation exercises on patients and healthy people in unstable conditions according to different sizes of balls (different levels), through which some conclusions were got that could improve the activity of some muscles in some sense. In addition, suspension exercises were also integrated in core exercise, that is, appending multiplanar as well as multijoint [1] meanwhile dynamic limb movement [2]. It demonstrated a good way to enhance surface electromyographic (sEMG) signal of some muscles. However, this study was conducted merely in a stable plane (the traditional core stability exercise). Maybe we could use others in the future.

The signal of sEMG has shown a good way to assess muscle activity, which is applied in sports, rehabilitation, and other fields. Analytical methods are conducted not only in time domain (integrated EMG, root-mean-square value, and zero-crossing point) [20] but also in frequency domain (median frequency and mean power frequency) [21]. Based on root-mean-square value, the maximum voluntary isometric contraction (MVIC) was the most used method to analyze the signal of sEMG activity [22] in time domain during the rehabilitation exercise, and researchers already drew some conclusions which were propitious to athletes, patients, and healthy people [13]. They showed that some exercises had some obvious effect for some muscles, but, for other muscles, other exercises [4, 6, 23] were needed. However, there were few studies in frequency domain about rehabilitation exercise. From these existing researches [10–12, 21, 24], we knew that median frequency was an important index to evaluate muscle fatigue. It can show a downward trend as time going on if the muscles present fatigue. While doing the traditional rehabilitation exercises, we can feel our muscle fatigue, but there were few studies about the muscle fatigue during the classic rehabilitation exercises. In this study, we used the median frequency to analyze the activity of selected muscles during four classic rehabilitation exercises. According to the rehabilitation therapists' proposal, each exercise holds on 1 minute. And the classic rehabilitation exercises are prone bridge, supine bridge, unilateral bridge, and bird-dog, respectively, which have been widely applied in research, rehabilitation, and daily exercise [6, 9, 13, 19, 21].

The purpose of this study was to investigate the fatigability differences by utilizing median frequency of sEMG and conclude the muscle activities while performing the given four exercises, where the slopes of median frequency were calculated according to the selected muscles in lower limb, abdominal, and back; at the same time, the different fatigability in the left and right sides of the same-named muscle was analyzed during the same rehabilitation exercises.

## 2. Methods

### 2.1. Participants

30 healthy graduate students (15 female and 15 male) from our university volunteered for this study. None of them has any pain in their body especially in the back, abdominal, and leg in the past one year when they perform the exercise or is allergic to electrodes. The subjects' characteristics were shown in Table 1. They were informed the details about the test and agreed to participant in this experiment before they were involved in this study.

### 2.2. Data Recording

All experiments were conducted in a quiet, bright, thermostatic laboratory. The sEMG signals were recorded using the 16-channel, wireless sEMG system (MyoMuscle, Noraxon USA, Inc.) which connected to a laptop computer. The surface electrodes we used were Disposable Ag/AgCl Electrodes which were made in Shanghai LITU Medical Appliances Limited Company.

**Figure 1:**
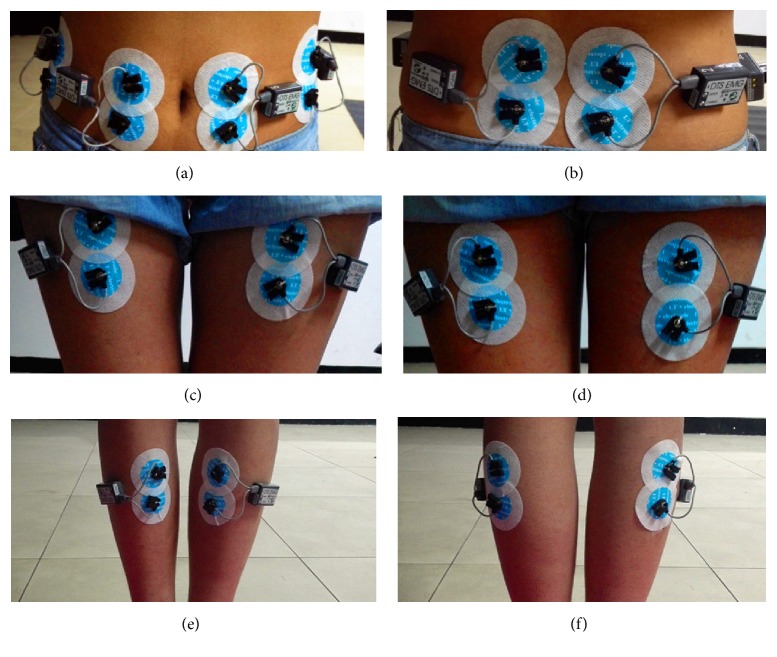
Electrode position of both left and right sides of muscles. (a) was the RA and EO muscles, (b) was ES muscle, (c) was RF muscle, (d) was BF muscle, (e) was AT muscle, and (f) was G muscle.

Prior to sticking the electrodes, the skin was cleared in the following two steps: firstly, using the sandpaper to abrade the skin and, secondly, cleaning the sticking area with alcohol of concentration 75%, which made the skin impedance less than 10 kΩ. When we placed the electrodes, we should pay attention to the interelectrode distance. It was about 20 mm. The electrodes should be parallel to the direction of muscle fibers [14, 25]. In this study, we collected a total of fourteen muscles of one body. They were rectus femoris (RF), biceps femoris (BF), anterior tibial (AT), gastrocnemius (G), rectus abdominis (RA), external oblique (EO), and erector spinae (ES) muscles [3, 6, 7, 10, 14, 15, 26] (both left and right sides muscles), respectively. The electrode positions were shown in Figures 1(a)–1(f).

So we collected fourteen-channel sEMG signals. Each channel was connected to two-circle electrodes. The electrodes were connected to a DTS sensor where the reference electrode was in this module. The sEMG signals were received through the TeleMyo DTS Desk Receiver and transmitted to the laptop computer.

**Figure 2:**
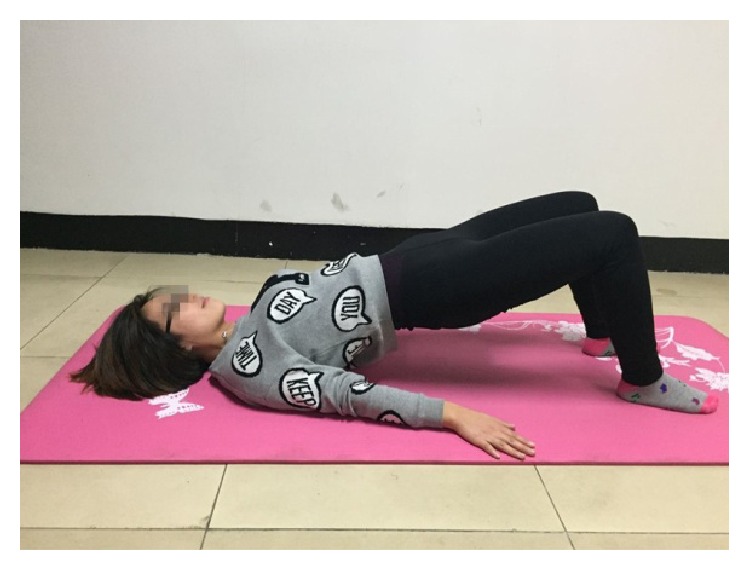
Supine bridge.

**Figure 3:**
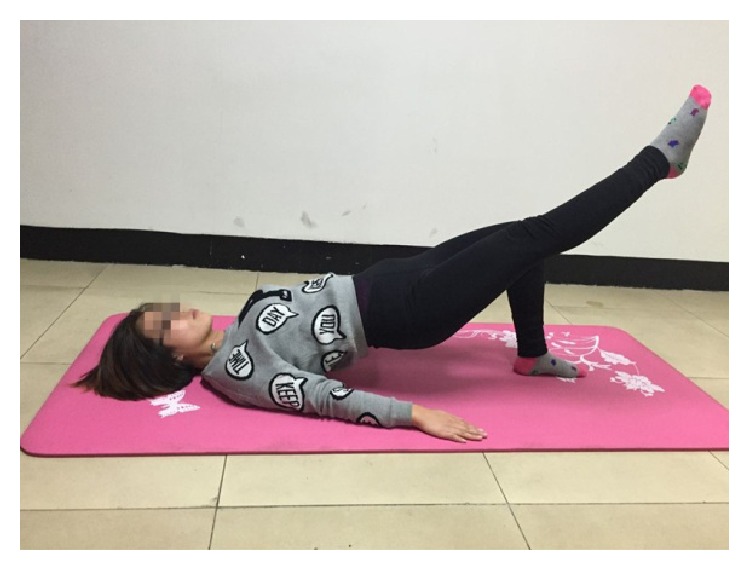
Unilateral bridge.

**Figure 4:**
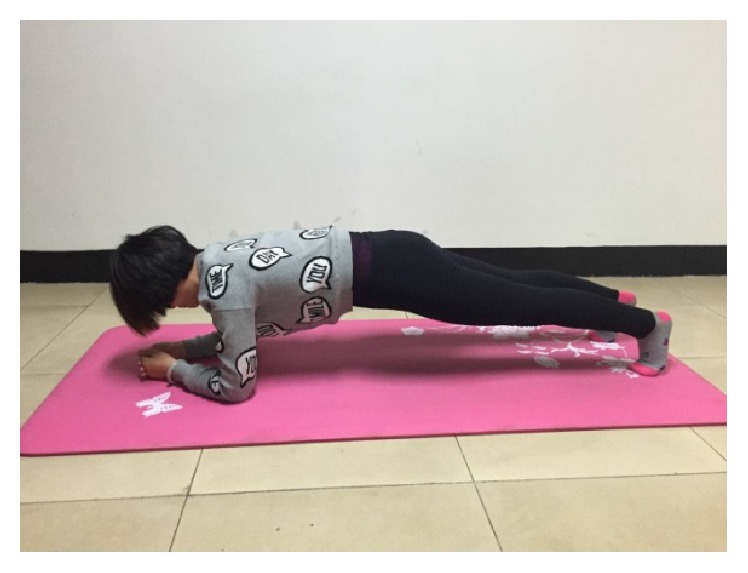
Prone bridge.

**Figure 5:**
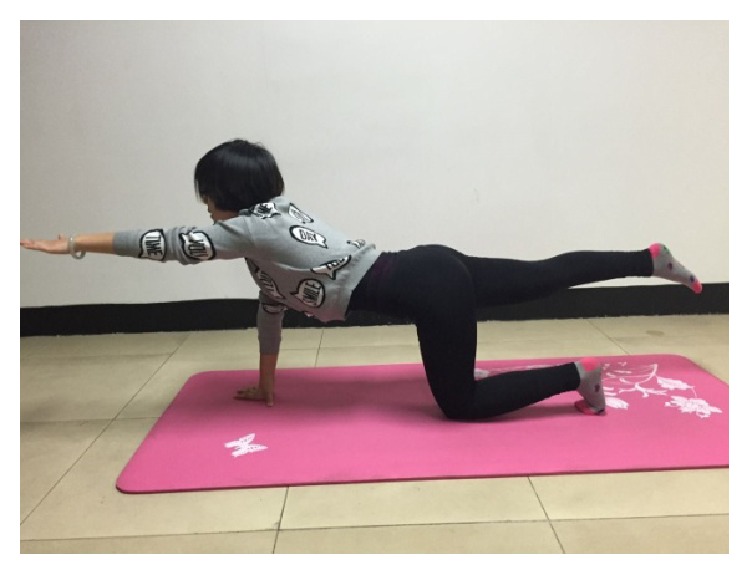
Bird-dog.

The four core stability exercises were shown in Figures 2–5.  Figure 2 showed the supine bridge. During this exercise, lifting the abdominal directly with the knees bending into 90°, the toes faced forward and the hands at sides on the floor. On the basis of the first case of supine bridge, lifting one leg (left or right leg) is random. The unilateral bridge was shown in Figure 3. Prone bridge was shown in Figure 4; only the elbows and toes popped up of the body and the body was parallel to the ground during this exercise. The last exercise, with the name of bird-dog, was shown in Figure 5. During this exercise, the subject lifted the left (right) arm and right (left) leg in neutral spine alignment, which faces the ground. The subject was asked to perform each exercise for one minute. A two-minute rest was given between two exercises for relaxation. The order of performing these exercises was random.

### 2.3. Data Processing and Analyzing

The raw sEMG signals were sampled at 1500 Hz, band-pass-filtered from 80 to 250 Hz using the finite impulse response (FIR) filters, and full-wave-rectified. Then root-mean-square (window of 50 ms) algorithm was used to smooth filtering of the sEMG signal. These signals were processed in time domain, which were operated in one-second step interval from the beginning to the end of the whole selected period. We used the median frequency (MF) of frequency fatigue report to track and analyze fatigue related changes in the neuromuscular recruitment. Changes in the frequency, median frequency slope, could be used to determine local fatigue [11, 24, 27].

The frequency fatigue reports were then saved as  .slk file, which could be opened and edited by Microsoft Excel. The MF values of all the same muscles on the same side of all subjects were placed in the same Excel table. A one-way ANOVA was used to analyze the differences between MF and its slope of left and right sides of the same-named muscles; besides MF and its slope of different muscles were also analyzed which were doing the same rehabilitation exercise. The confidence interval is set to 0.05.

## 3. Results

All the results were obtained from using the MF, comparing the MF slope of the seven muscles during the different exercises, including the left or the right side of the same-named muscle in one exercise. From the statistical results under all exercises, every muscle showed a certain consistency about the muscle fatigue. We chose the muscles which over 50% of subjects showed fatigue to analyze and the muscles were shown in Table 2. “*∗*” represents the numbers of subjects' muscles showing fatigue that were over 50% of all subjects during the rehabitation exercise, and the numbers of subjects were also shown in Table 2.

### 3.1. Median Frequency Analysis

**Figure 6:**
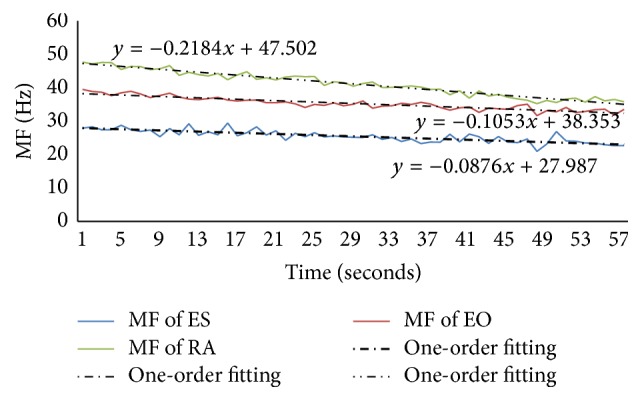
The MF and its one-order fitting for ES, EO, and RA in the prone bridge exercise.

**Figure 7:**
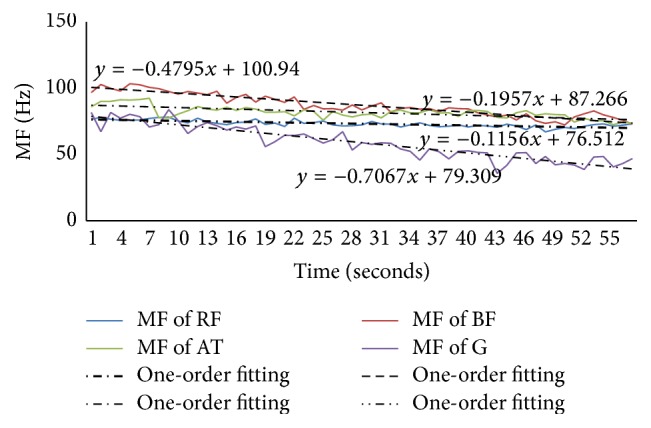
The MF and its one-order fitting for RF, BF, AT, and G muscles in the exercise of prone bridge.

**Figure 8:**
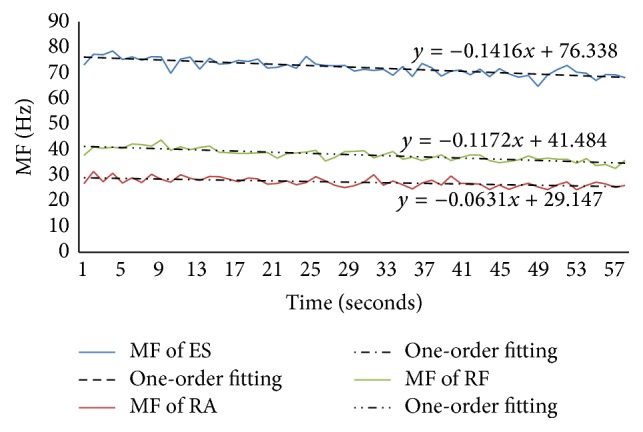
The MF and its one-order fitting for ES, RA, and RF muscles in the exercise of supine bridge.

**Figure 9:**
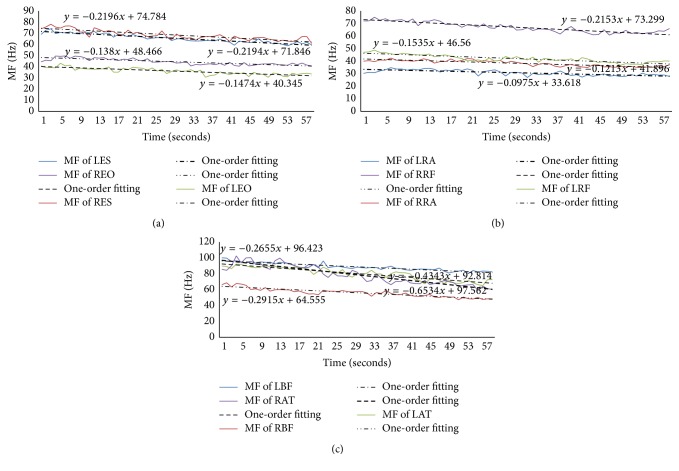
(a) The MF of left and right sides of ES and EO muscles and their one-order fitting during the unilateral bridge (lifting right leg). (b) The MF of left and right sides of RA and RF muscles and their one-order fitting during the unilateral bridge (lifting right leg). (c) The MF of left and right sides of BF and AT muscles and their one-order fitting during the unilateral bridge (lifting right leg).

Figures 6–9 showed the MF and its one-order fitting for muscles during the four rehabilitation exercises and the data were the average MF of different times in which the subjects' muscles showed fatigue, and the numbers were shown in Table 2.

During the prone bridge, the most was subjects' RA muscle, where about 91% of subjects revealed fatigue, the fewest was the ES muscle where about 54% of subjects showed fatigue, and the others were in this range. We analyzed all muscles (the left side) in this exercise. The MF of low back muscles were shown in Figure 6, and the MF of lower limb muscles were shown in Figure 7.

Figures 6 and 7 presented the values of MF and its one-order fitting of low back muscles (ES, EO, and RA muscles) and lower limb muscles (RF, BF, AT, and G muscles) during the period of prone bridge test, respectively. The values of MF all showed declining trend in different degrees though fluctuated according to the time. From the one-order fitting, the absolute value of the slope of the three low back muscles, RA, was the greatest, the less was EO, and the least was ES. However, for lower limb muscles, the absolute value of the slope of G muscle's MF was larger than other muscles. Compared with Figure 6, the absolute value of the slope of MF for G muscle was higher than ES muscle and the MF of lower limb muscle were greater than the low back muscle.

For the supine bridge, there are three muscles where the numbers of subjects showing fatigue were over 50%: RF muscle (65% of subjects), ES muscle (52% of subjects), and RA muscle (50% of subjects), respectively, so they were put in a figure to analyze. We chose the left side muscle to analyze as well as prone bridge, which was shown in Figure 8.

Figure 8 showed the value of MF and its one-order fitting of ES, RA, and RF muscles during the supine bridge exercise. It showed that ES muscle was the most active; however the RA muscle was the least active.

Based on supine bridge, one situation was lifting right leg upward and the other was lifting left leg upward in unilateral bridge exercise. We chose the first one to analyze, as shown in Figure 9. In this exercise, 76% of subjects' BF muscle and 57% of subjects' AT muscle showed fatigue, G muscle was below 50%, and the others were in this range.

Figure 9 showed the MF of ES, EO, RA, RF, BF, and AT muscles and their one-order fitting. The absolute value of slope showed that AT muscle was the most active, especially in the left side, the least active one was RA muscle, and the rest were in the range.

**Figure 10:**
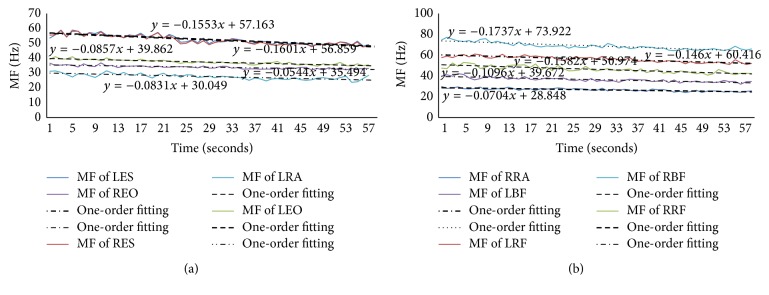
(a) The MF of ES and EO muscles both left and right sides and left side of RA muscle as well as their one-order fitting in the bird-dog exercise. (b) The MF of RF and BF muscles both left and right sides and right side of RA muscle as well as their one-order fitting in the bird-dog exercise.

In the exercise of bird-dog, there were 78% of subjects' EO muscle and 53% of subjects' RF muscle showed fatigue, the AT and G muscles of below 50% of subjects showed fatigue, and the others were between 53% and 78% of subjects. We still chose one condition to analyze, which was lifting left arm and right leg. The results were shown in Figure 10.

Figure 10 exhibited the MF of ES, EO, RA, RF, and BF muscles both in left side and in right side as well as their one-order fitting. The absolute value of slope showed that the right side of BF muscle was the most active; however the right side of EO was the least active among these muscles during bird-dog exercise.

### 3.2. Median Frequency Slope

**Figure 11:**
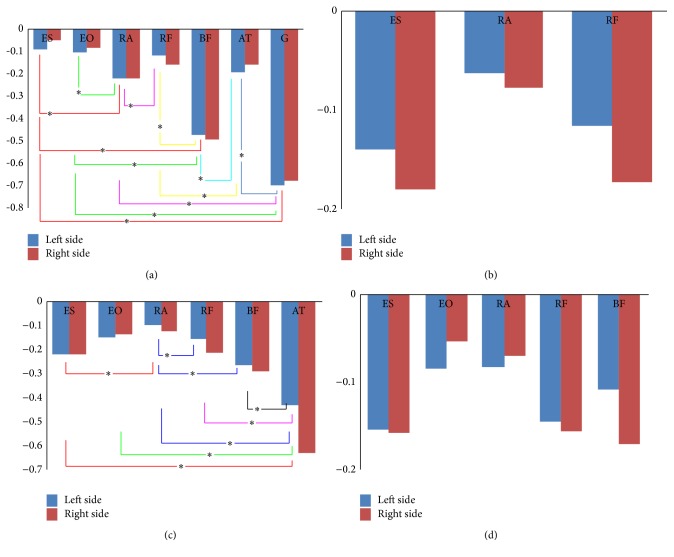
The MF slope of some muscles during the four rehabilitation exercises. The horizontal axis represents the muscles of left side and right side; the vertical coordinate represents the median frequency slope. (a) showed the results of prone bridge; (b) showed the results of supine bridge; (c) showed the results of unilateral bridge (lifting the right leg); and (d) showed the results of bird-dog exercise (lifting the left arm and right leg). “*∗*” represents that there was significant difference between the two muscles.

Figures 11(a)–11(d) demonstrated the MF slope of the muscles (according to Section 3.1) of both left and right sides during the four rehabilitation exercises.

Figure 11 exhibited the median frequency slope of muscles which showed fatigue. There was no significant difference (*P* > 0.05) between left and right sides in the same-named muscles about muscle fatigue during the four rehabilitation exercises. From Figures 11(b) and 11(d) we knew that there was no significant difference (*P* > 0.05) between any two muscles fatigue which we analyzed in the supine bridge and bird-dog rehabilitation exercises. We knew that the most fatigue muscle was G muscle and the least fatigue one was ES muscle in the prone bridge exercise from Figure 11(a). There were significant differences (*P* < 0.05) between the two muscles of the seven muscles which were using “*∗*” to label significant difference in Figure 11(a). Similarly, the condition of muscle fatigue significant differences was in unilateral bridge (lifting the right leg) which was shown in Figure 11(c). Compared with supine bridge (Figure 11(b)), the unilateral bridge (lifting right leg) caused more muscle fatigue, and the level of muscle fatigue increased. AT muscle was the most fatigue muscle, and the right side (the lifting side) was more fatigue than the left side (the supported side) apparently. The results of the last exercise, bird-dog, was shown in Figure 11(d), which caused most muscle fatigue, but the difference between each other was not obvious (*P* > 0.05). However, there has been little difference between the left side (the supported side) and the right side (the lifting side).

## 4. Discussion

The purpose of this study was to investigate the activity of the low back muscles and the selected lower muscles during the four rehabilitation exercises. Based on the previous researches, MF and its slope of the sEMG signals [10, 24, 27–29] have been applied to analyze the muscle activity, especially the muscle fatigue. This study used the sEMG signal characteristics (median frequency and its slope) in frequency domain to analyze the seven muscles activity during four rehabilitation exercises. The main results of this study were the different muscles activity during the four traditional rehabilitation exercises, especially the selected lower muscles' activity which used the frequency domain methods.

The four rehabilitation exercises have been investigated in previous studies [13, 19]. For distinguishing and drawing further conclusions from these studies, we have used frequency domain methods (MF and its slope) and analyzed the lower limb muscles.

The prone bridge (Figure 4) contributed to seven muscles fatigue which can be learned from Figure 11; that is to say, this exercise can adequately stimulate much more muscles than others. From the previous studies [13, 16], they have found that EO and RA muscles have higher activity level than the ES muscle. From Figure 11(a), we can get that the MF slope of EO and RA muscles was greater than ES muscles, and there was significant difference between RA muscle and ES muscles' MF slope. Ekstrom et al. [13] had tested the vastus medialis obliquus and Hamstrings muscles' activity in prone bridge and found that their activity level was inferior to lumbar and low back muscles. However, we have found that BF and G muscles' fatigue level was higher than ES, EO, and RA muscles, and AT as well as RF muscles have a similar fatigue level to the ES, EO, and RA muscles. That means prone bridge exercise is suitable for training back leg and abdominal muscles.

Comparing unilateral bridge (left side was supported side and right side was lifted side) (Figure 11(c)) to supine bridge (Figure 11(b)), the unilateral bridge exerted much more muscles' fatigue, and the level was higher too, which was similar to previous studies [9, 13, 17, 19]. Czaprowski et al. [19] and Ekstrom et al. [23] concluded that the supine bridge induced the lowest muscle activity comparing to the prone bridge exercise, bird-dog exercise, and these exercises on unstable plane, which we got from Figure 11. Although there was no significant difference (*P* > 0.05) between the left side (supported side) and right side (lifted side) of the same-named lower limb muscles, we can found that the right side muscles' fatigue was higher than the left side muscles' fatigue. Feldwieser et al. [9] reported that the difference of unilateral bridge exercise on both sides of the body muscle activity exist because the lifted side muscle has to counteract the gravity of lifting side to keep the trunk and pelvis in a bridged condition, so we knew the reason for unilateral bridge exercise of the lifted side' muscles activity enhancement. We also found that ES muscle' fatigue level was higher than EO and RA muscles, which was the same as previous investigations [13, 19].

During the bird-dog exercise (Figure 5), which includes lifting the right leg and left arm (i.e., the left side was the supported side and the right side was the lifted side), five muscles showed fatigue in over 50% of subjects. Okubo et al. [16] had found that there was no obvious difference in the ES, EO, and RA muscles' activity level as well as these muscles' left and right sides, and in this study we also found there was no significant difference (*P* > 0.05) in the left and right sides of the five muscles (ES, EO, RA, RF, and BF muscles) for MF slope and between these five muscles which can be obtained from Figure 11(d). The MF slope of the five muscles was not particularly high; namely, the bird-dog exercise would not make these muscles much fatigue. We can also get that, as reported in previous studies [13, 19, 23], the muscles (EO, RA, ES, and some lower limb muscles) activity was moderate in the bird-dog exercise. Thus we can regard the bird-dog exercise as a moderate exercise.

As discussed above, firstly, the prone bridge stimulate much muscles which can train abdominal [13, 19] and BF as well as G muscles especially; secondly, combining the unilateral bridge and supine bridge would increase the muscle activity in order to train our muscles much better [9]; thirdly, the bird-dog exercise can be considered as a moderate exercise which could be useful for developing muscle endurance [13, 23]. The limitations of this study were as follows: we have not compare activity level of the same muscle in different rehabilitation exercises and these differences between the female and male. In addition, we have not divided the muscle fatigue into different levels.

## 5. Conclusions

Different activities were found during the four rehabilitation exercises among the selected seven muscles. The prone bridge caused most subjects' fatigue including all seven muscles, especially the G and RA muscles. The supine bridge only arouses three muscles (ES, RA, and RF muscles) where over 50% of subjects exhibited fatigue, and the level of fatigue was tiny. The unilateral bridge exercises also caused most muscle fatigue except for G muscle, especially for AT and ES muscles. The bird-dog exercise made most muscles fatigue and relatively moderate.

For healthy people, we should do prone bridge exercise to train abdominal and calf muscles, do unilateral bridge exercise for back muscles, and do bird-dog exercise to balance our whole body muscles; then our physical quality would be improved.

## Figures and Tables

**Table 1:** Subjects characteristics (mean ± SD).

Gender	Age (year)	Height (cm)	Weight (kg)
Female	24.0 ± 2.0	160.0 ± 5.0	50.0 ± 5.0
Male	24.0 ± 2.0	176.0 ± 5.0	65.0 ± 10.0

**Table 2:** Relationship between muscle fatigue and rehabilitation exercise.

Exercises	Muscles						
	ES	EO	RA	RF	BF	AT	G
Prone bridge	*∗* (16)	*∗* (24)	*∗* (27)	*∗* (19)	*∗* (19)	*∗* (17)	*∗* (20)

Supine bridge	*∗* (16)		*∗* (15)	*∗* (20)			

Unilateral bridge (lifting right leg)	*∗* (20)	*∗* (21)	*∗* (22)	*∗* (18)	*∗* (23)	*∗* (17)	

Bird-dog (lifting left arm and right arm)	*∗* (16)	*∗* (23)	*∗* (20)	*∗* (16)	*∗* (20)		

## References

[1] Mok N. W., Yeung E. W., Cho J. C., Hui S. C., Liu K. C., Pang C. H. (2015). Core muscle activity during suspension exercises. *Journal of Science and Medicine in Sport*.

[2] Park H.-J., Oh D.-W., Kim S.-Y. (2014). Effects of integrating hip movements into bridge exercises on electromyographic activities of selected trunk muscles in healthy individuals. *Manual Therapy*.

[3] Pereira L. M., Marcucci F. C. I., de Oliveira Menacho M., Garanhani M. R., Lavado E. L., Cardoso J. R. (2011). Electromyographic activity of selected trunk muscles in subjects with and without hemiparesis during therapeutic exercise. *Journal of Electromyography and Kinesiology*.

[4] Escamilla R. F., Lewis C., Bell D. (2010). Core muscle activation during Swiss ball and traditional abdominal exercises. *Journal of Orthopaedic & Sports Physical Therapy*.

[5] Sakeran H., Mahmud M. F., Jamal M. I. EMG analysis of upper and lower rectus abdominis during exercises performed on and off a swiss ball.

[6] Imai A., Kaneoka K., Okubo Y. (2010). Trunk muscle activity during lumbar stabilization exercises on both a stable and unstable surface. *Journal of Orthopaedic & Sports Physical Therapy*.

[7] Lehman G. J., Hoda W., Oliver S. (2005). Trunk muscle activity during bridging exercises on and off a Swissball. *Chiropractic & Osteopathy*.

[8] Stevens V. K., Bouche K. G., Mahieu N. N., Coorevits P. L., Vanderstraeten G. G., Danneels L. A. (2006). Trunk muscle activity in healthy subjects during bridging stabilization exercises. *BMC Musculoskeletal Disorders*.

[9] Feldwieser F. M., Sheeran L., Meana-Esteban A., Sparkes V. (2012). Electromyographic analysis of trunk-muscle activity during stable, unstable and unilateral bridging exercises in healthy individuals. *European Spine Journal*.

[10] Marshall P. W., Murphy B. A. (2005). Core stability exercises on and off a Swiss ball. *Archives of Physical Medicine and Rehabilitation*.

[11] Van Damme B., Stevens V., Van Tiggelen D., Perneel C., Crombez G., Danneels L. (2014). Performance based on sEMG activity is related to psychosocial components: differences between back and abdominal endurance tests. *Journal of Electromyography and Kinesiology*.

[12] Sung P. S., Lammers A. R., Danial P. (2009). Different parts of erector spinae muscle fatigability in subjects with and without low back pain. *The Spine Journal*.

[13] Ekstrom R. A., Donatelli R. A., Carp K. C. (2007). Electromyographic analysis of core trunk, hip, and thigh muscles during 9 rehabilitation exercises. *Journal of Orthopaedic & Sports Physical Therapy*.

[14] Farahpour N., Ghasemi S., Allard P., Saba M. S. (2014). Electromyographic responses of erector spinae and lower limb's muscles to dynamic postural perturbations in patients with adolescent idiopathic scoliosis. *Journal of Electromyography and Kinesiology*.

[15] Feger M. A., Donovan L., Hart J. M., Hertel J. (2014). Lower extremity muscle activation during functional exercises in patients with and without chronic ankle instability. *American Academy of Physical Medicine and Rehabilitation*.

[16] Okubo Y., Kaneoka K., Imai A. (2010). Electromyographic analysis of transversus abdominis and lumbar multifidus using wire electrodes during lumbar stabilization exercises. *Journal of Orthopaedic & Sports Physical Therapy*.

[17] Kavcic N., Grenier S., McGill S. M. (2004). Quantifying tissue loads and spine stability while performing commonly prescribed low back stabilization exercises. *Spine*.

[18] Imai A., Kaneoka K., Okubo Y., Shiraki H. (2014). Effects of two types of trunk exercises on balance and athletic performance in youth soccer players. *The International Journal of Sports Physical Therapy*.

[19] Czaprowski D., Afeltowicz A., Gebicka A. (2014). Abdominal muscle EMG-activity during bridge exercises on stable and unstable surfaces. *Physical Therapy in Sport*.

[20] Dong-mei W., Xin S., Zhi-cheng Z., Zhi-jiang D. (2010). Feature collection and analysis of surface electromyography signals. *Zhongguo Zuzhi Gongcheng Yanjiu yu Linchuang Kangfu*.

[21] Hongjun Y. (2002). Functional status of muscle in surface electromyography. *Chinese Journal of Clinical Rehabilitation*.

[22] Hibbs A. E., Thompson K. G., French D. N., Hodgson D., Spears I. R. (2011). Peak and average rectified EMG measures: which method of data reduction should be used for assessing core training exercises?. *Journal of Electromyography and Kinesiology*.

[23] Ekstrom R. A., Osborn R. W., Hauer P. L. (2008). Surface electromyographic analysis of the low back muscles during rehabilitation exercises. *Journal of Orthopaedic and Sports Physical Therapy*.

[24] Beneck G. J., Baker L. L., Kulig K. (2013). Spectral analysis of EMG using intramuscular electrodes reveals non-linear fatigability characteristics in persons with chronic low back pain. *Journal of Electromyography and Kinesiology*.

[25] Hermens H. J., Freriks B., Disselhorst-Klug C., Rau G. (2000). Development of recommendations for SEMG sensors and sensor placement procedures. *Journal of Electromyography and Kinesiology*.

[26] Xu G., Xu M. (1998). Electromyographic analysis of lower limb muscle for bridging exercise. *Journal of Rehabilitation Medicine*.

[27] Ali M., Bandpei M., John Watson M. (2001). Electromyographic power spectral analysis of the paraspinal muscles: reliability study. *Physiotherapy*.

[28] Elfving B., Dedering Å. (2007). Task dependency in back muscle fatigue—correlations between two test methods. *Clinical Biomechanics*.

[29] Farina D., Gazzoni M., Merletti R. (2003). Assessment of low back muscle fatigue by surface EMG signal analysis: methodological aspects. *Journal of Electromyography and Kinesiology*.

